# Draft Genome Sequence of the Earliest *Cronobacter sakazakii* Sequence Type 4 Strain, NCIMB 8272

**DOI:** 10.1128/genomeA.00782-13

**Published:** 2013-09-26

**Authors:** Naqash Masood, Karen Moore, Audrey Farbos, Sumyya Hariri, Konrad Paszkiewicz, Ben Dickins, Alan McNally, Stephen Forsythe

**Affiliations:** Pathogen Research Group, School of Science and Technology, Nottingham Trent University, Clifton Lane, Nottingham, United Kingdoma; Wellcome Trust Biomedical Informatics Hub, Biosciences, University of Exeter, Exeter, United Kingdomb

## Abstract

The *Cronobacter sakazakii* clonal lineage defined as sequence type 4 (ST4) is associated with severe cases of neonatal meningitis and persistence in powdered infant formula. For genome sequencing of the earliest deposited culture collection strain of *Cronobacter sakazakii* ST4, we used the strain NCIMB 8272, originally isolated from milk powder in 1950.

## GENOME ANNOUNCEMENT

The *Cronobacter* genus is associated with severe human infections such as meningitis, septicemia, and necrotizing enterocolitis (1). A multilocus sequence typing scheme has been established for the genus and over 200 sequence types (ST) have been defined (1). Previous studies of *Cronobacter* isolates collected over a 35-year period across 6 countries have revealed a strong association of *Cronobacter sakazakii* sequence type 4 (ST4) with neonatal meningitis (2). In addition, *C. sakazakii* ST4 has frequently (up to 24% of strains) been isolated from powdered infant formula (PIF) manufacturing plants as well as from PIF (3, 4). Therefore, efforts to improve understanding of *C. sakazakii* ST4 are warranted to gain insight regarding its prevalence in manufacturing plants and infant formula and its pathogenicity. In this study, we sequenced *C. sakazakii* NCIMB 8272 as it is the oldest strain in international culture collections, having been isolated from milk powder in 1950 (1). This strain can thus be compared with more recently isolated and sequenced *Cronobacter* species (5).

*C. sakazakii* NCIMB 8272 DNA was extracted from 1-day cultures using a GenElute bacterial genome kit (Sigma-Aldrich) and sequenced using an Illumina HiSeq2500 system. A total of 7,102,990 high-quality paired-end reads 150 bp in length, with 30-fold coverage, were generated. *De novo* assembly was performed using Velvet (6). Further annotation was performed using the SEED-based automated annotation system provided by the RAST Server (7).

The genome sequence of *C. sakazakii* NCIMB 8272 was 4,583,199 bp in length with a G+C content of 56.8%. The draft genome was distributed in 104 contigs with 4,259 coding sequences (CDS) and 97 RNAs.

The CDS included genes associated with iron acquisition, stress response, heavy metal resistance (arsenic, silver cobalt, zinc, and cadmium), and phages. Several virulence-associated traits such as adhesion and sialic acid utilization were also determined. These have previously been described in *Cronobacter* (5).

### Nucleotide sequence accession number.

The genome sequences of *C. sakazakii* NCIMB 8272 have been deposited in GenBank under the accession number AWFW00000000.

## References

[1] Joseph S Sonbol H Hariri S Desai P McClelland M Forsythe SJ 2012 Diversity of the *Cronobacter* genus as revealed by multilocus sequence typing. J. Clin. Microbiol. 50:3031–3039 22785185 10.1128/JCM.00905-12 PMC3421776

[2] Hariri S Joseph S Forsythe SJ 2013 Predominance of *Cronobacter sakazakii* ST4 clonal complex strains in *Cronobacter* neonatal meningitis infections in US 2011. Emerg. Infect. Dis. 19:175–177 23260316 10.3201/eid1901.120649 PMC3557988

[3] Müller A Stephan R Fricker-Feer C Lehner A 2013 Genetic diversity of *Cronobacter sakazakii* isolates collected from a Swiss infant formula production facility. J. Food Prot. 76:883–887 23643134 10.4315/0362-028X.JFP-12-521

[4] Sonbol H Joseph S McAuley C Craven H Forsythe SJ 2013 Multilocus sequence typing of *Cronobacter* spp. from powdered infant formula and milk powder production factories. Int. Dairy J. 30:1–7

[5] Joseph S Desai P Ji Y Cummings CA Shih R Degoricija L Rico A Brzoska P Hamby SE Masood N Hariri S Sonbol H Chuzhanova N McClelland M Furtado MR Forsythe SJ 2012 Comparative analysis of genome sequences covering the seven *Cronobacter* species. PLOS One 7:e49455 23166675 10.1371/journal.pone.0049455 PMC3500316

[6] Zerbino DR Birney E 2008 Velvet: algorithms for *de novo* short read assembly using de Bruijn graphs. Genome Res. 18:821–829 18349386 10.1101/gr.074492.107 PMC2336801

[7] Aziz RK Bartels D Best AA DeJongh M Disz T Edwards RA Formsma K Gerdes S Glass EM Kubal M Meyer F Olsen GJ Olson R Osterman AL Overbeek RA McNeil LK Paarmann D Paczian T Parrello B Pusch GD Reich C Stevens R Vassieva O Vonstein V Wilke A Zagnitko O 2008 The RAST server: Rapid annotations using subsystems technology. BMC Genomics 9:75. 10.1186/1471-2164-9-75 18261238 PMC2265698

